# Strain, disinfectant, concentration, and contact time quantitatively impact disinfectant efficacy

**DOI:** 10.1186/s13756-018-0340-2

**Published:** 2018-04-03

**Authors:** Alyssa M. West, Peter J. Teska, Caitlinn B. Lineback, Haley F. Oliver

**Affiliations:** 1 Department of Food Science, Purdue University, 745 Agriculture Mall Drive, West Lafayette, IN 47907 USA; 2 Diversey Inc., Charlotte, NC 28273 USA

**Keywords:** Disinfectant, Label, Concentration, MRSA, *Pseudomonas*

## Abstract

**Background:**

Transmission of healthcare-associated infections caused by antibiotic- and multi-drug resistant (MDR) pathogens (e.g. Methicillin-resistant *Staphylococcus aureus* (MRSA), *Pseudomonas aeruginosa*) are a major concern in patient care facilities. Disinfectant usage is critical to control and prevent pathogen transmission, yet the relationships among strain, disinfectant type, contact time, and concentration are not well-characterized. We hypothesized that there would be significant differences in disinfectant efficacy among clinically relevant strains under off-label disinfectant conditions, but there would be less no differences among at registered label use concentrations and contact times. The purpose of this study was to quantify the effect of disinfectant concentration and contact time on the bactericidal efficacy of clinically relevant strains of *S. aureus* and *P. aeruginosa*.

**Methods:**

Accelerated hydrogen peroxide (AHP), quaternary ammonium compounds (Quat), and sodium hypochlorite were tested at label and reduced contact times and concentrations against four MDR *P. aeruginosa* strains and four MRSA strains. Quantitative EPA method MB-25-02 was used to measure disinfectant efficacy reported as log_10_ reduction.

**Results:**

Both off-label disinfectant concentrations and contact times significantly affected efficacy of all disinfectants tested. Bactericidal efficacy varied among MRSA and *P. aeruginosa* strains.

**Conclusions:**

The quantitative disinfectant efficacy method used highlights the inter-strain variability that exists within a bacterial species. It also underscores the need for a disinfectant validation method that takes these variances into account.

## Background

An estimated 722,000 healthcare-associated infections (HAIs) resulted in ~ 75,000 deaths in the United States in 2011 [1]. Methicillin-resistant *Staphylococcus aureus* (MRSA) is a multi-drug resistant (MDR) pathogen that caused an estimated 55,000 healthcare-associated invasive infections in the United States in 2014 [2]. *Pseudomonas aeruginosa* is a leading cause of hospital-acquired pneumonia and the number one cause of wound infections in burn unit patients [3]. The emergence of multi-drug and antibiotic- resistant bacteria has led to an increased effort to improve cleaning and disinfection procedures in healthcare facilities.

Environmental contamination has been recently recognized as a contributing factor to HAIs. A study by Bhalla et al. found that healthcare workers frequently acquired nosocomial pathogens on their hands after coming into contact with environmental surfaces [4]. Another study tracked contamination of soft surfaces using tracer viruses [5]. This study showed that the tracer viruses easily spread from volunteers’ hands to multiple soft surfaces around the healthcare facility [5]. Multiple studies have also shown that healthcare-associated pathogens can persist on surfaces in the environment for long periods of time [6, 7]. Therefore, proper disinfecting procedures are crucial in helping prevent the transmission of HAIs.

The use of Environmental Protection Agency (EPA)-registered disinfectant products is encouraged by both the CDC and EPA [8]. Users of EPA-registered products must abide by the specific concentration and contact times listed on the label in order for the disinfectant to achieve a five-log reduction. Some disinfectants need a full 10 min of surface contact time for the product to be effective. Such a long contact time can be hard to achieve in healthcare facilities due to the time-pressured environment [8]. Therefore, it is important to understand how off-label use of EPA-registered disinfectants may affect the efficacy of the product.

Disinfectant usage is a key part of environmental control to prevent HAI transmission in healthcare environments. However, the relationship between disinfectant efficacy and MDR pathogens has not been well-characterized by current disinfectant efficacy methodologies. The objective of this study was to examine the effect of disinfectant concentration and contact time on bactericidal efficacy against *S. aureus* and *P. aeruginosa*. We hypothesized that there would be quantifiable differences in disinfectant efficacy among clinically relevant strains under off-label disinfectant conditions, but there would not be significant differences among strains at defined label use concentrations and contact times.

## Methods

### Disinfectants and bacterial strains used in this study

In this study, we evaluated the disinfectant efficacy of AHP, Quat, and sodium hypochlorite disinfectants using EPA standard operating procedure MB-25-02 [9]. In this study, we used *S. aureus* ATCC CRM-6538 and *P. aeruginosa* ATCC 15442 as control strains, as well as four MDR *P. aeruginosa* strains (Table 1) and four MRSA strains (Table 2). Oxivir 1 (EPA 70627–56, Diversey Inc., Charlotte, NC) was selected to represent AHP disinfectants; it contained 0.5% hydrogen peroxide. The Quat-based disinfectant used was Virex Tb (EPA 76027–24, Diversey Inc., Charlotte, NC), which contained 0.105% dimethyl benzyl ammonium chloride and 0.105% dimethyl ethyl benzyl ammonium chloride. Avert (EPA 70627–75, Diversey Inc., Charlotte, NC) contained 1.2% sodium hypochlorite. All three disinfectants were packaged at ready-to-use concentrations. The disinfectant label contact time was one minute for Oxivir 1 and three minutes for Virex TB. The disinfectant label contact time for Avert as a sporicidal agent against *Clostridium difficile* was four minutes, but as a bactericidal agent the label contact time was one minute; Avert was tested at both label contact times at label concentration.

**Table 1:** Characteristics of *Staphylococcus aureus* strains used in study

Species of Microorganism	ATCC Strain Name	PFGE Type	SCCmec Type	*pvl* gene	Isolation Source
Methicillin-resistant *Staphylococcus aureus*	ATCC BAA-1717	USA 300	Type IV	Positive	Adolescent patient with severe sepsis
Methicillin-resistant *Staphylococcus aureus*	ATCC BAA-1761	USA 100	Type II	Negative	Human Subject
Methicillin-resistant *Staphylococcus aureus*	ATCC BAA-1720	USA 200	Type II	Negative	Hospital acquired
Methicillin-resistant *Staphylococcus aureus*	ATCC BAA-1754	USA 600	Type IV	Negative	Human Subject
*Staphylococcus aureus*	ATCC CRM-6538	–	–	–	Human Lesion

**Table 2:** Characteristics of *Pseudomonas aeruginosa* strains used in study

Species of Microorganism	ATCC Strain Name	Isolation Source	Antibiotic Resistance
*Pseudomonas aeruginosa*	ATCC BAA-2108	Sputum Sample, human	AMX, AMP, CFZ, CXM, CAE, CTX, FOX, IPM, NIT, TGC, and TMP
*Pseudomonas aeruginosa*	ATCC BAA-2112	Sputum Sample, human	AMX, AMP, CFZ, CLO, CXM, CAE, CTX, FOX, CRO, NIT, TGC, and TMP
*Pseudomonas aeruginosa*	ATCC BAA-2113	Sputum Sample, human	AMX, AMP, CFZ, CLO, CXM, CAE, CTX, FOX, CRO, NIT, TGC, and TMP
*Pseudomonas aeruginosa*	ATCC BAA-2114	Sputum Sample, human	AMX, AMP, TZP, CFZ, CLO, CXM, CAE, CTX, FOX, CRO, NIT, TGC, and TMP
*Pseudomonas aeruginosa*	ATCC 15442	Animal room water bottle	–

Briefly, stainless steel coupons were inoculated with a soil load composed of 67.3% bacterial culture, 8.7% yeast, 19.2% mucin, and 4.8% bovine serum albumin. Each disinfectant was applied to inoculated coupons at a defined concentration and contact time; the surviving bacterial load was recovered after application of neutralizing buffer. Recovered bacteria were filtered onto 0.2 μm pore membrane disc filters subsequently plated onto tryptic soy agar; colonies were counted after incubation at 37 °C for 24–48 h.

### Bactericidal efficacy of disinfectants at label use, reduced concentrations, and reduced contact times

To determine the effect of concentration on bactericidal efficacy, three disinfectant concentrations (50%, 75%, and 100% of label concentrations) with a constant contact time of one minute for Oxivir 1, three minutes for Virex Tb, and four minutes for Avert were measured. Disinfectants were at ready-to-use concentrations and were diluted using hard water (according to EPA protocol) to reach lower concentration levels. Experiments were performed using stainless steel coupons at approximately 22 °C. Each organism and concentration combination consisted of three disinfectant-treated coupons (technical replicates) and four phosphate buffered saline (PBS)-treated control coupons. The same set of controls was used when testing two disinfectants against the same organism with the same variable treatment. Each treatment was independently repeated three times for Oxivir 1 and Avert and five times for Virex Tb. A prior study by Hong et al. found that this method is more variable for Quat-based disinfectants, thus more replicates were conducted to minimize potential error [10]. Bactericidal efficacy was measured at four disinfectant contact times (30 s, 1 min, 2 min, and 3 min) at label concentration to determine the effect of varying disinfectant contact times. To determine the reduction of bacteria in every strain, time, and concentration permutation, the log10 bacterial count of each experimental treatment was compared to the corresponding control bacterial counts (control coupons were exposed to PBS instead of the disinfectant).

### Statistical analyses

Statistical Analysis System (SAS) (version 9.4, SAS Institute Inc., Cary, NC) was used for all analyses. All data were transformed to log10 reduction values for analyses. All replicates were run independently for each disinfectant. One-way ANOVA was used to determine if, under label use conditions, bacterial strain type was significantly correlated to log10 reduction values (α = 0.05). Tukey Honest Significant Difference (HSD) test was used to determine strain-specific differences. A generalized linear mixed model (GLMM) was used to assess the impact of off-label contact time, concentration, and their interactions for each disinfectant independently (α = 0.05). Bacterial strain type was considered a random variable to determine the effect of contact time and concentration on disinfectant efficacy irrespective of strain type. An additional GLMM was used to determine specific differences in log10 reduction among strains exposed to each disinfectant; least squares means with Holm-Tukey adjustment for multiple comparisons were used to determine significant differences among the strains at off-label conditions (α = 0.05).

## Results

### Disinfectant usage at label conditions has varied impact on bactericidal efficacy among strains

**Fig. 1:**
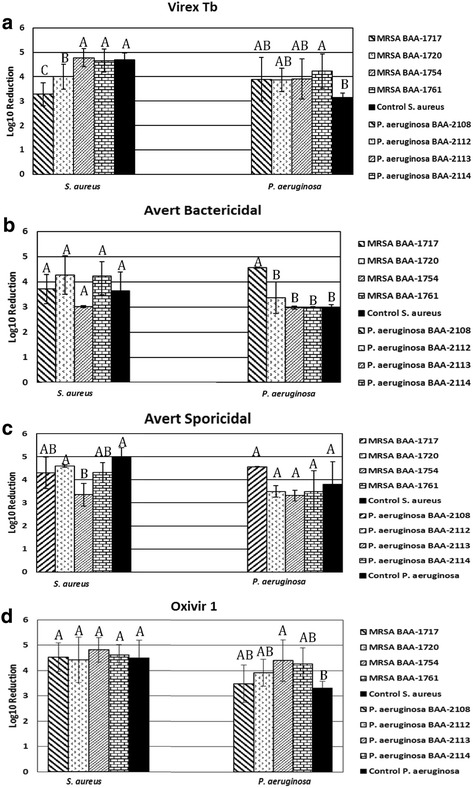
Log_10_ reduction values of each bacterial strain for a given disinfectant used at label conditions. Letters above bars indicate statistical grouping and significant differences among strains. **a**: Bactericidal efficacy of Virex Tb (Quat-based) against various MRSA and *P. aeruginosa* strains at label conditions (three-minute contact time). **b**: Bactericidal efficacy of Avert (Chlorine-based) against various MRSA and *P. aeruginosa* strains at bactericidal label conditions (one minute contact time). **c**: Bactericidal efficacy of Avert (Chlorine-based) against various MRSA and *P. aeruginosa* strains at *C. difficile* sporicidal label conditions (four-minute contact time). **d**: Bactericidal efficacy of Oxivir 1 (AHP-based) against various MRSA and *P. aeruginosa* strains at label conditions (one minute contact time)

Overall, there were significant differences in disinfectant efficacy at label conditions among strains of both *P. aeruginosa* and MRSA (ANOVA with Tukey HSD; *P* = 0.0132 and *P* < 0.0001), respectively (Fig. 1A). Virex Tb, at label conditions, was significantly less effective against MRSA ATCC BAA-1720 and BAA-1717 compared to all other MRSA strains tested (*P* < 0.05). Virex Tb was significantly more effective against *P. aeruginosa* strain ATCC BAA-2114 than the control *P. aeruginosa* strain (P < 0.05).

There were no significant differences among MRSA strains at the bactericidal (one min) label contact time (Fig. 1B). At sporicidal label contact time (four min), there were significant differences in disinfectant efficacy among the strains (Fig. 1C). The *S. aureus* control strain and MRSA ATCC BAA-1720 both had significantly higher disinfectant susceptibility compared to MRSA ATCC BAA-1754 (ANOVA with Tukey HSD; *P* < 0.05). Avert had a significantly higher disinfectant efficacy against *P. aeruginosa* ATCC BAA-2108 compared to all other *P. aeruginosa* strains at bactericidal contact time (P < 0.05). There were no significant differences in disinfectant efficacy among the *P. aeruginosa* strains at sporicidal contact time.

There was a significant difference in disinfectant efficacy among the strains of *P. aeruginosa* (ANOVA with Tukey HSD; *P* = 0.023) (Fig. 1D). Specifically, Oxivir 1 was more effective against *P. aeruginosa* ATCC BAA-2113 than the *P. aeruginosa* control strain (*P* < 0.05). There were no significant differences in disinfectant efficacy among MRSA strains at label conditions.

### Reduced disinfectant concentrations have varied effects on bactericidal efficacy among strains

**Fig. 2:**
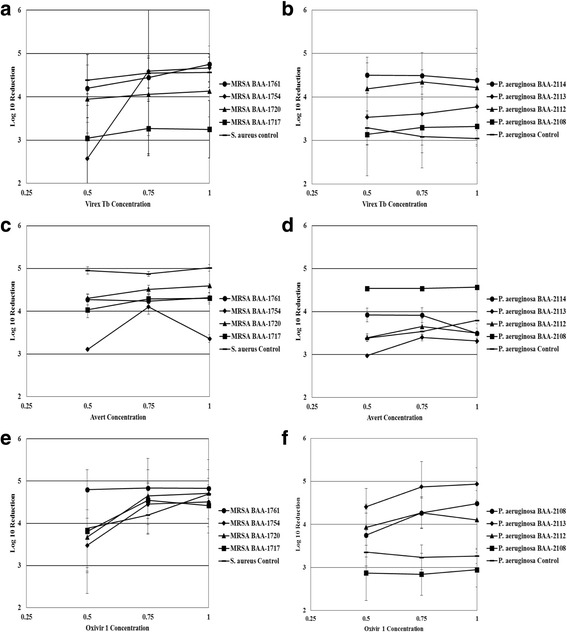
Effect of varying disinfectant concentration on the bactericidal efficacy of three disinfectants at label concentration. **a**: Bactericidal efficacy of Virex Tb (Quat-based) against various MRSA strains. **b**: Bactericidal efficacy of Virex Tb (Quat-based) against various *P. aeruginosa* strains. **c**: Bactericidal efficacy of Avert (Chlorine-based) against various MRSA strains. **d**: Bactericidal efficacy of Avert (Chlorine-based) against various *P. aeruginosa* strains. **e**: Bactericidal efficacy of Oxivir 1 (AHP-based) against various MRSA strains. Panel F: Bactericidal efficacy of Oxivir 1 (AHP-based) against various *P. aeruginosa* strains

Disinfectant concentration had an overall significant effect on the efficacy of Virex Tb (GLMM; *P* < 0.0001). The interaction between strain and disinfectant concentration was also significant (P < 0.0001). Virex Tb was more effective against MRSA ATCC BAA-1761 than both MRSA ATCC BAA-1717 and BAA-1754 (P < 0.0001) (Fig. 2A). Virex Tb was significantly more effective against *P. aeruginosa* ATCC BAA-2114 than *P. aeruginosa* ATCC BAA-2108 (GLMM with Holm-Tukey Correction; P_adj_ < 0.0001), *P. aeruginosa* ATCC BAA-2113 (P_adj_ = 0.0078), and the control strain (P_adj_ < 0.0001) at varying disinfectant concentration (Fig. 2B).

Overall, disinfectant concentration significantly affected the efficacy of Avert (GLMM; *P* = 0.0209). Specifically, concentrations less than defined label use resulted in increased recovery of bacteria. Avert was significantly more effective against the *S. aureus* control strain than MRSA ATCC BAA-1717 (GLMM with Holm-Tukey Correction; P_adj_ = 0.0434) and MRSA ATCC BAA-1754 (P_adj_ < 0.0001) at varying disinfectant concentrations (Fig. 2C). Avert was also significantly more effective against *P. aeruginosa* ATCC BAA-2108 than the control strain (P_adj_ = 0.0029), *P. aeruginosa* ATCC BAA-2112 (P_adj_ = 0.0015), *P. aeruginosa* ATCC BAA-2113 (P_adj_ < 0.0001), and *P. aeruginosa* ATCC BAA-2114 (P_adj_ = 0.0257) under varying disinfectant concentrations (Fig. 2D).

Disinfectant concentration had a significant effect on the efficacy of Oxivir 1 (*P* = 0.0027). There was no significant difference in disinfectant efficacy with varying disinfectant concentrations among MRSA strains (Fig. 2E). Oxivir 1 was significantly more effective against *P. aeruginosa* ATCC BAA-2112 (GLMM with Holm-Tukey Correction; P_adj_ = 0.0105), *P. aeruginosa* ATCC BAA-2113 (P_adj_ < 0.0001), *P. aeruginosa* ATCC BAA-2114 (P_adj_ = 0.0051) than the control strain at varying disinfectant concentrations (Fig. 2F). Oxivir 1 was significantly less effective against *P. aeruginosa* ATCC BAA-2108 (P_adj_ < 0.0001) than *P. aeruginosa* ATCC BAA-2112, BAA-2113, and BAA-2114 at varying disinfectant concentrations (Fig. 2F).

### Reduced disinfectant contact times have varied effects on bactericidal efficacy among strains

**Fig. 3:**
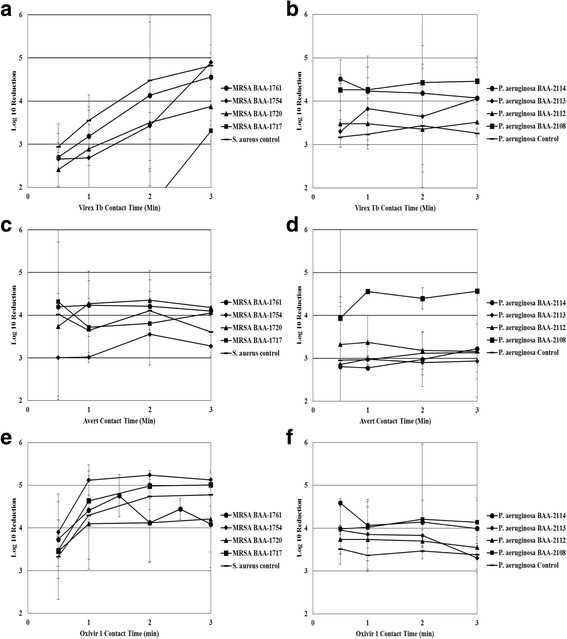
Effect of varying disinfectant contact time on bactericidal efficacy of three disinfectants at label concentration. **a**: Bactericidal efficacy of Virex Tb (Quat-based) against various MRSA strains. **b**: Bactericidal efficacy of Virex Tb (Quat-based) against various *P. aeruginosa* strains. **c**: Bactericidal efficacy of Avert (Chlorine-based) against various MRSA strains. **d**: Bactericidal efficacy of Avert (Chlorine-based) against various *P. aeruginosa* strains. **e**: Bactericidal efficacy of Oxivir 1 (AHP-based) against various MRSA strains. Panel F: Bactericidal efficacy of Oxivir 1 (AHP-based) against various *P. aeruginosa* strains

Disinfectant contact time had an overall significant effect on the efficacy of Virex Tb (GLMM; *P* < 0.0001). The interaction between strain and disinfectant contact time was also significant (P < 0.0001). Virex Tb was significantly more effective against the control *S. aureus* strain than MRSA ATCC BAA-1717 (GLMM with Holm-Tukey Correction; P_adj_ < 0.0001), MRSA ATCC BAA-1720 (P_adj_ = 0.0007), and MRSA ATCC BAA-1754 (P_adj_ *=* 0.0372) at varying disinfectant contact times (Fig. 3A). The disinfectant was also more effective against MRSA ATCC BAA-1761 than both MRSA ATCC BAA-1717 and BAA-1754 (P < 0.0001) (Figs. 3A). Virex Tb was significantly more effective against *P. aeruginosa* ATCC BAA-2114 than *P. aeruginosa* ATCC BAA-2112 (P_adj_ = 0.0001), *P. aeruginosa* ATCC BAA-2113 (P_adj_ = 0.0155), and the control strain (P_adj_ < 0.0001), at varying disinfectant contact times (Fig. 3B).

Avert was significantly more effective against MRSA ATCC BAA-1717 (P_adj_ = 0.0215) and MRSA ATCC BAA-1720 (P_adj_ = 0.0035) than MRSA ATCC BAA-1754 at varying disinfectant contact times (Fig. 3C). Avert was significantly more effective against *P. aeruginosa* ATCC BAA-2108 than the other four *P. aeruginosa* ATCC strains (GLMM with Holm-Tukey Correction; all P_adj_ < 0.0001) (Fig. 3D).

Overall, disinfectant contact time (GLMM; *P* = 0.0328) had a significant effect on the efficacy of Oxivir 1. Oxivir 1 was significantly more effective against MRSA ATCC BAA-1754 than MRSA ATCC BAA-1720 (P_adj_ = 0.0099) and BAA-1761 (P_adj_ = 0.0008) (Fig. 3E). Oxivir 1 was significantly more effective against *P. aeruginosa* ATCC BAA-2108 (P_adj_ = 0.0081) and *P. aeruginosa* ATCC BAA-2114 (P_adj_ = 0.0015) than the control strain at varying disinfectant contact times (Fig. 3F).

## Discussion

In this study, we tested three disinfectants under label and off-label use condition on strains with varying antibiotic resistance profiles using EPA method MB-25-02 [7]. Using this quantitative EPA method, we found significant, quantifiable differences in disinfectant efficacy among strains of *S. aureus* and *P. aeruginosa* at label and off-label use conditions and that these differences varied by disinfectant.

### There are significant differences in disinfectant efficacy among strains tested under label-use conditions

We found significant differences among strains at disinfectant label use conditions; the results varied based on disinfectant. For example, Virex Tb (quat-based) was significantly less effective against MRSA ATCC BAA-1720 yet Avert (chlorine-based) was significantly more effective at sporicidal conditions against the strain. Differences among strains were observed for all disinfectants at label-use conditions. Current EPA testing methods to register a healthcare disinfectant (for use on hard non-porous surfaces) requires testing against the two control strains used in this study [11]; it does not evaluate different strains. One of the specific testing methods that can be used for registering disinfectants is AOAC Use-Dilution method [12]. Positive and negative results are determined through a visual inspection for the presence or absence of turbidity (indicating microbial growth). The AOAC Use-Dilution method is qualitative, whereas the method used in this study (MB 25–02) is quantitative. Our data suggest that further research is warranted to determine if the disinfectant efficacy testing for EPA registration should be re-evaluated to include multiple strains.

### Efficacy of reduced concentration and contact time is strain dependent

Overall, our results showed that disinfectant efficacy varied by strain under off-label conditions. Our data showed that off-label use of a disinfectant product does lead to reduced efficacy. Per United States federal law regarding EPA-registered products, all applicable label instructions must be followed for proper disinfectant use and to achieve full efficacy [13]. A review by Schabrun and Chipchase in 2006 noted that “equipment used in the non-critical setting is less likely to have standard cleaning protocols than equipment used in the critical setting” [14]. A study by Davis looked at blood pressure cuffs as a potential source of cross-contamination in hospitals [15]. This study concluded that that these non-critical medical devices needed more vigilant disinfection procedures and called into question if the time-constrained emergency nurses were best suited for the disinfection role [15]. Furthermore, the CDC’s Guidelines for Disinfection and Sterilization in Healthcare Facilities notes numerous factors that may influence a disinfectant’s contact time or concentration, and therefore efficacy [13]. Using disinfectants to clean complex medical devices can be challenging. It can be difficult to expose the internal channels of a medical device to the disinfectant for the label contact time, especially as internal air pockets will interfere with this process [13]. In this study, we used disinfectants at ready-to-use concentrations. Concentrated products are common and are typically diluted with water; water hardness can reduce disinfection efficacy. Efficacy can be lowered by the formation of insoluble precipitates due to the presence of divalent cations in hard water [13]. Neither the AOAC Use-Dilution Testing method or the EPA method we used in this study take this real-world problem into account. Both methods use specially prepared water that won’t interfere with the disinfectant efficacy testing [9, 12].

Although our study highlighted the importance of disinfectant contact time and concentration to achieve efficacy, it is still not well-defined what level of efficacy is needed for a healthcare environment to be considered hygienic. EPA Standard Operating Procedures are very specific on the soil level, surface type, and bacterial strains to be used when doing efficacy testing. This makes it difficult to translate efficacy from lab testing to actual environmental disinfection. This study was limited to ten strains as well, warranting more work to be done.

## Conclusion

In this study, we found differences in disinfectant efficacy amongst strains at label and off-label conditions. Further, we found that concentration and contact time significantly affect disinfectant efficacy of three disinfectants in different ways. The consequences of off-label use of disinfectants is demonstrated in this study. This study underscores the variability of disinfectant efficacy within a bacterial genus. Our data suggests additional strains and additional test methods should be investigated to better understand inter-strain variability.

## Abbreviations

AHP: Accelerated Hydrogen Peroxide
EPA: Environmental Protection Agency
MDR: Multi-Drug Resistant
MRSA: Methicillin Resistant *Staphylococcus aureus*
PBS: Phosphate Buffered Saline
Quat: Quaternary Ammonium Compounds
